# The Role of Macrophage Polarization and Ferroptosis in the Progression of Liver Fibrosis

**DOI:** 10.1155/cjgh/1200073

**Published:** 2025-11-27

**Authors:** Jeri Nobia Purnama, Mohammad Ghozali, Desak Made Malini, Ratu Safitri

**Affiliations:** ^1^ Graduate School, Padjadjaran University, Bandung, Indonesia, unpad.ac.id; ^2^ Department of Biomedical Sciences, Faculty of Medicine, Padjadjaran University, Jatinangor, Bandung, 45363, West Java, Indonesia, unpad.ac.id; ^3^ Department of Biology, Faculty of Mathematics and Natural Sciences, Padjadjaran University, Jatinangor, Bandung, 45363, West Java, Indonesia, unpad.ac.id

## Abstract

Liver fibrosis is a pathological condition marked by excessive extracellular matrix accumulation, potentially leading to cirrhosis. Macrophages play a vital role in regulating inflammatory responses, facilitating tissue repair, and orchestrating extracellular matrix remodeling through their differentiation into proinflammatory and anti‐inflammatory phenotypes. Hepatic stellate cell activation and the progression of fibrosis have been linked to ferroptosis, a controlled cell death process triggered by iron and characterized by lipid peroxidation. This review explores the interaction between ferroptosis and macrophage polarization in iron‐overload‐induced liver fibrosis. It examines how ferroptosis intensifies inflammatory and fibrotic processes through macrophage activity and identifies key macrophage marker proteins involved. Understanding this interplay offers novel therapeutic insights targeting macrophage polarization to mitigate liver fibrosis, particularly in conditions such as hemochromatosis and chronic transfusion‐dependent disorders.

## 1. Introduction

Liver fibrosis results from chronic liver injury and inflammation, leading to scar tissue formation. It is characterized by excessive extracellular matrix (ECM) deposition, which, if untreated, progresses to cirrhosis and liver failure [1]. Various factors, including chronic hepatitis, alcohol overconsumption, and iron overload, contribute to fibrosis development [2]. The immune system, particularly macrophages, plays a pivotal role in regulating inflammation and tissue remodeling during this process [3]. Liver fibrosis due to chronic iron overload represents a significant clinical burden, especially in transfusion‐dependent patients such as those with β‐thalassemia. In a recent multicenter analysis of mortality among thalassemia patients, gastrointestinal‐related deaths accounted for 4.9% of all deaths, with liver cirrhosis comprising 30.1% of those gastrointestinal causes, underscoring the fatal potential of progressive iron‐induced liver damage [4]. Furthermore, studies estimate that up to 65%–75% of β‐thalassemia major patients develop hepatic fibrosis, correlating with liver iron concentration and transfusional burden. The 2022 EASL Clinical Practice Guidelines emphasize the importance of monitoring iron burden and early intervention using chelation to prevent liver complications. Despite these advances, the molecular mechanisms that mediate the transition from iron overload to fibrosis, particularly the roles of ferroptosis and macrophage polarization, remain incompletely understood [5].

Macrophages exhibit plasticity and can transition between M1 and M2 phenotypes based on environmental stimuli [6]. M1 macrophages, activated by interferon‐γ (IFN‐γ) and lipopolysaccharide (LPS), produce proinflammatory cytokines such as TNF‐α and IL‐6. In contrast, M2 macrophages, induced by IL‐4 and IL‐13, facilitate tissue repair and resolution of inflammation [7]. The dynamic balance between these phenotypes influences liver fibrosis progression.

Liver fibrosis is caused by several factors, one of which is excess iron accumulation in the liver [8]. The condition of excess iron can be found in someone who has hereditary or secondary hemochromatosis metabolic disorders, such as thalassemia patients. Thalassemia patients are at risk of liver fibrosis caused by the accumulation of iron in the body due to reduced erythrocyte age and the frequency of blood transfusions [9]. Increased iron accumulation due to erythrocyte rupture and blood transfusions in the liver, which has a role in storing iron reserves in the form of ferritin, can trigger oxidative stress and chronic [10, 11]. The continued increase in inflammation in hepatocyte cells can lead to cellular death, known as ferroptosis. Ferroptosis is a term for iron‐dependent cell death characterized by an imbalance between increased lipid peroxidation and deficiency of the antioxidants glutathione peroxidase 4 (GPX4) and glutathione (GSH) [12]. Ferroptosis plays a vital role in damaging liver parenchymal cells and providing harmful signals that activate the immune response. Hepatocyte cell death leads to the release of damage signals that trigger macrophage recruitment and polarization. Increased lipid peroxidation and iron accumulation can trigger inflammatory reactions that worsen fibrosis conditions through interactions between macrophages and liver stellate cells [13].

This article aims to review recent literature on the link between iron overload‐induced ferroptosis and M1/M2 macrophage polarization and its contribution to the progression of liver fibrosis. It also explores how ferroptosis affects macrophage activity in the liver and the mechanisms underlying the polarization process. This article will also identify macrophage marker proteins that can be used to evaluate M1/M2 polarization in the context of iron accumulation‐induced liver fibrosis. Thus, this article is expected to serve as a reference for further research focusing on developing therapeutic strategies based on macrophage polarization regulation as a potential approach in the treatment of liver fibrosis.

## 2. Mechanism of Liver Fibrosis and Role of the Immune System

### 2.1. Pathogenesis of Liver Fibrosis

Hepatic fibrosis is the first stage of liver failure, leading to liver cirrhosis in response to wound healing from repeated liver injury [14]. Liver fibrosis is characterized by the abnormal formation of collagen produced by myofibroblasts and increased ECM deposition [15]. Liver fibrosis represents an early stage of liver failure, characterized by excessive ECM deposition due to repeated injury. Myofibroblasts, primarily derived from hepatic stellate cells (HSCs), drive fibrogenesis through collagen production [16]. Persistent fibrosis can advance to cirrhosis, hepatocellular carcinoma (HCC), and liver dysfunction. The resolution of fibrosis often involves the deactivation of HSCs, emphasizing the importance of targeting these cells in antifibrotic therapies.

### 2.2. Role of the Immune System

Macrophages are central regulators of fibrosis, influencing HSC activation via cytokines and growth factors. Proinflammatory macrophages (M1) secrete TNF‐α and IL‐1β, amplifying inflammation and promoting HSC activation [17]. In contrast, M2 macrophages release IL‐10, facilitating tissue repair by producing matrix metalloproteinases (MMPs) and tissue inhibitors of metalloproteinases (TIMPs) through the STAT3 and TGF‐β/Smad pathways. Studies highlight M1 macrophages as primary drivers of fibrosis progression. For instance, Handa et al. [18] demonstrated that iron‐enriched diets exacerbate fibrosis via increased M1 macrophage polarization through the CCL2 activation pathway. Additional studies indicate that modulating macrophage activity via signaling inhibitors, such as Notch pathway inhibitors, can suppress fibrosis. Natural compounds like resveratrol and gallic acid also exhibit antifibrotic properties by regulating TGF‐β/Smad3 signaling [19].

Mechanisms related to macrophage regulation in the process of liver fibrosis are very complex, involving various pathways and signaling molecules. Bansal & Chamroonkul [20], in their study, stated that inhibition of the Notch has an effect in regulating the expression levels of M1 and M2 to reduce HSC cell activation and collagen production. In addition, studies using resveratrol, gallic acid, or ferulic acid showed inhibition by reducing inflammation and liver fibrosis through the TGF‐β/Smad3 signaling pathway in mice induced with CCL4 [21] signaling [19]. Other cases, such as liver fibrosis caused by iron overload conditions in the body, were also reported by Shendge et al. [22], who state that there is an effect of white mulberry administration on reducing liver fibrosis through regulation of MAPKs (ERK, JNK, and p38).

## 3. Ferroptosis and Its Role in Liver Fibrosis

### 3.1. Regulation of Ferroptosis

One form of iron‐dependent cell death called ferroptosis is distinguished by oxidative stress and lipid damage [12]. This process disrupts cellular homeostasis by depleting key antioxidants such as GPX4 and GSH, leading to an imbalance in redox status. It is initiated by excessive iron accumulation, which triggers the Fenton reaction and generates reactive oxygen species (ROS), ultimately causing lipid membrane damage. If left uncontrolled, this cascade can lead to hepatocyte death and the progression of fibrosis [23].

Transferrin (Tf) transports iron ions (Fe^3+^) into cells through interaction with Tf receptors on hepatocyte membranes. Upon entry into endosomes, Fe^3+^ is reduced to Fe^2+^ by the STEAP3 enzyme, forming a labile iron pool that can participate in the Fenton reaction, generating ROS. The accumulation of ROS triggers uncontrolled lipid peroxidation, which contributes to the ferroptosis process. GSH‐dependent GPX4 activity inhibits lipid peroxidation; however, failure in this antioxidant system will increase lipid peroxidation, ROS, and, ultimately, ferroptosis.

**Figure 1:**
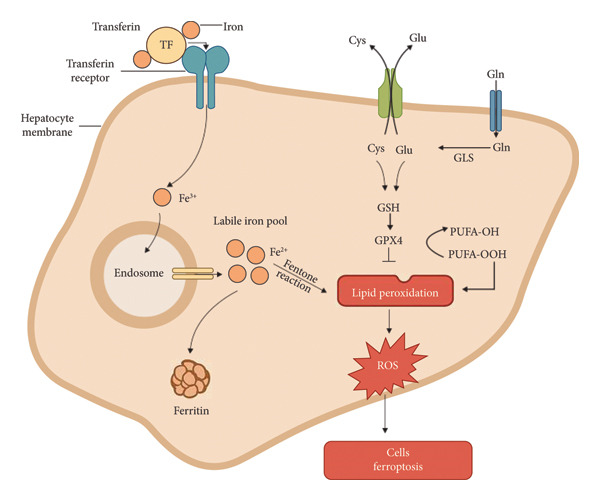
Schematic of the mechanism of ferroptosis in hepatocytes through iron accumulation.

Excess iron can also disrupt the function of the antioxidant system, leading to an imbalance of oxidant and antioxidant systems in the body [24]. The decreased activity of the enzyme GPX4, which plays a role in protecting cells from lipid peroxidation, is one of the effects of the development of the ferroptosis process. Ferroptosis is influenced by glutathione homeostasis (GSH) and the enzyme GPX4 [25]. GSH is synthesized through cysteine and glutamate transport mediated by the glutamate–cystine antiporter (Xc‐). GPX4 plays a role in inhibiting lipid peroxidation by converting PUFA‐OOH (unsaturated fatty acid peroxide) into a nonreactive form of PUFA‐OH [11]. When GPX4 cannot function properly, such as in GSH depletion, lipid peroxides will accumulate, resulting in an increase in ROS and ferroptosis. The Fenton reaction in the body is triggered by the presence of catalytic Fe2+ ions due to iron overload conditions in the body. The Fenton reaction produces free radicals that directly attack lipids and cause peroxidation of polyunsaturated fatty acids (PUFAs) (Figure 1), which contributes to the cellular death process.

One key regulator of intracellular iron homeostasis and ferroptosis is nuclear receptor coactivator 4 (NCOA4), which mediates ferritinophagy, the selective autophagic degradation of ferritin. NCOA4 binds to the ferritin heavy chain and facilitates its delivery to lysosomes, resulting in the release of free iron into the cytosol. This process increases the labile iron pool, enhancing ROS generation through the Fenton reaction and thereby sensitizing cells to ferroptosis. Recent studies also suggest that excessive NCOA4‐mediated ferritinophagy contributes to GPX4 inactivation, making cells more vulnerable to lipid peroxidation and cell death [26].

### 3.2. Role of Ferroptosis in Liver Fibrosis

**Figure 2:**
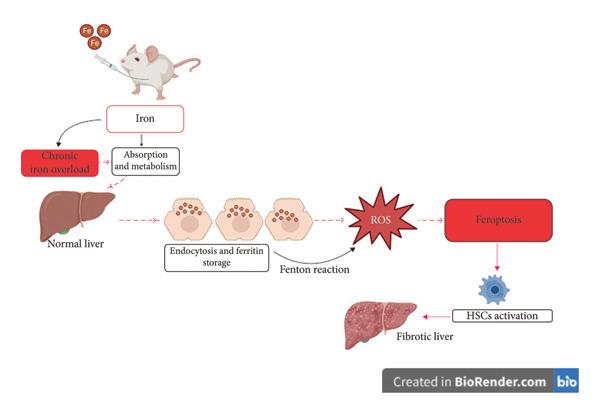
Illustration of the mechanism by which chronic iron overload contributes to liver fibrosis through ferroptosis. Iron accumulation improves reactive oxygen species (ROS) production via the Fenton reaction, triggering ferroptosis and subsequent activation of hepatic stellate cells (HSCs), leading to fibrotic liver progression.

Iron overload, common in thalassemia and hereditary hemochromatosis, accelerates fibrosis by intensifying ferroptosis‐induced inflammation. Research by Mohamed et al. [27] found that thalassemia patients with elevated ferritin levels exhibited increased fibrosis risk, particularly in those with hepatitis C virus (HCV) co‐infection. Fragkou et al. further demonstrated that liver cirrhosis occurs when hepatic iron concentrations exceed 250 μmol/g. Hepatocyte cell death through ferroptosis triggers the release of damage‐associated molecular patterns (DAMPs), activating Kupffer cells, which in turn promote HSC activation and fibrosis (Figure 2). Studies indicate that targeting ferroptosis in hepatocytes and Kupffer cells could serve as a therapeutic strategy to mitigate fibrosis progression.

Fragkou et al. [9], in their article, stated that when iron levels in the liver exceed 250 mol/g, liver cirrhosis is likely to occur. Several studies have also shown that iron overload increases the risk of liver fibrosis through increased HSC cell activation and increased TGF‐β expression in mice [28]. Research conducted by Ramm et al. [29] has demonstrated a connection between elevated α‐SMA protein expression and collagen deposition in hemochromatosis patients and iron levels in the liver. Similar results were also shown in a rat model of hemochromatosis, where increased HSC cell proliferation was associated with the level of α‐SMA expression and col3a1 protein expression [30, 31]. Beyond hepatocytes, Kupffer cells and HSCs play a central role in iron‐induced liver fibrosis. Kupffer cells, the resident macrophages of the liver, are highly sensitive to iron accumulation and oxidative stress. Under iron overload, ferroptotic death of Kupffer cells leads to the release of DAMPs, such as HMGB1, ATP, and mitochondrial DNA (mtDNA) [32]. These molecules activate Toll‐like receptors (TLRs) and inflammasome signaling in neighboring immune and non‐immune cells, amplifying the profibrotic microenvironment. DAMP‐mediated signaling can stimulate HSC activation, promoting their transdifferentiation into collagen‐producing myofibroblasts. HSCs themselves may also undergo ferroptosis, with paradoxical outcomes. In acute settings, ferroptosis of activated HSCs may help resolve fibrosis. However, in chronic iron overload, persistent ROS and lipid peroxidation can trigger stress adaptation in HSCs, leading to enhanced proliferation and ECM deposition. This dual role of ferroptosis in HSCs, both as a potential suppressor and promoter of fibrosis, warrants further exploration in disease models [33].

In pathological conditions, hepatocytes that undergo ferroptosis release danger or death signals that can activate macrophage cells in the liver (Kupffer cells) to secrete proinflammatory cytokines such as TNF‐α and IL‐1β in response to these signals. Activated Kupffer cells cause HSC cell activation and develop into liver fibrosis. Research conducted by Sui et al. [34] showed that HSC cells induced ferroptosis using RAS‐selective lethal 3 (RSL3) or elastin‐caused cell death. Similar treatments also showed increased expression of plasminogen activator inhibitor‐1 as well as increased phosphorylation of c‐JUN and luciferase protein activator one activity, indicating the role of ferroptosis in activating HSC cells in the development of liver fibrosis [13]. Modulation of ferroptosis in hepatocytes, Kupffer cells, and HSC cells suggests a potential therapeutic approach to liver fibrosis.

## 4. Macrophage Interaction and Ferroptosis in Liver Fibrosis

### 4.1. Macrophage Polarization

Macrophages are immune cells that play an important role in the immune system [3]. Macrophages are derived from precursor cells in the bone marrow and serve as the first line of protection against infection and foreign particles [35]. Macrophages are found in various body tissues and can detect, phagocytize, and destroy pathogens as well as dead or damaged cells. The ability of macrophages to adapt to signals from their microenvironment allows them to have a functional phenotype. Although macrophage phenotypes are traditionally classified into proinflammatory (M1) and anti‐inflammatory (M2) types, current evidence supports that macrophage polarization exists along a dynamic spectrum of activation states. These states are shaped by a complex network of microenvironmental signals, including IFN‐γ, LPS, IL‐4, IL‐13, and TGF‐β, resulting in intermediate or mixed phenotypes with overlapping functions [36].

**Figure 3:**
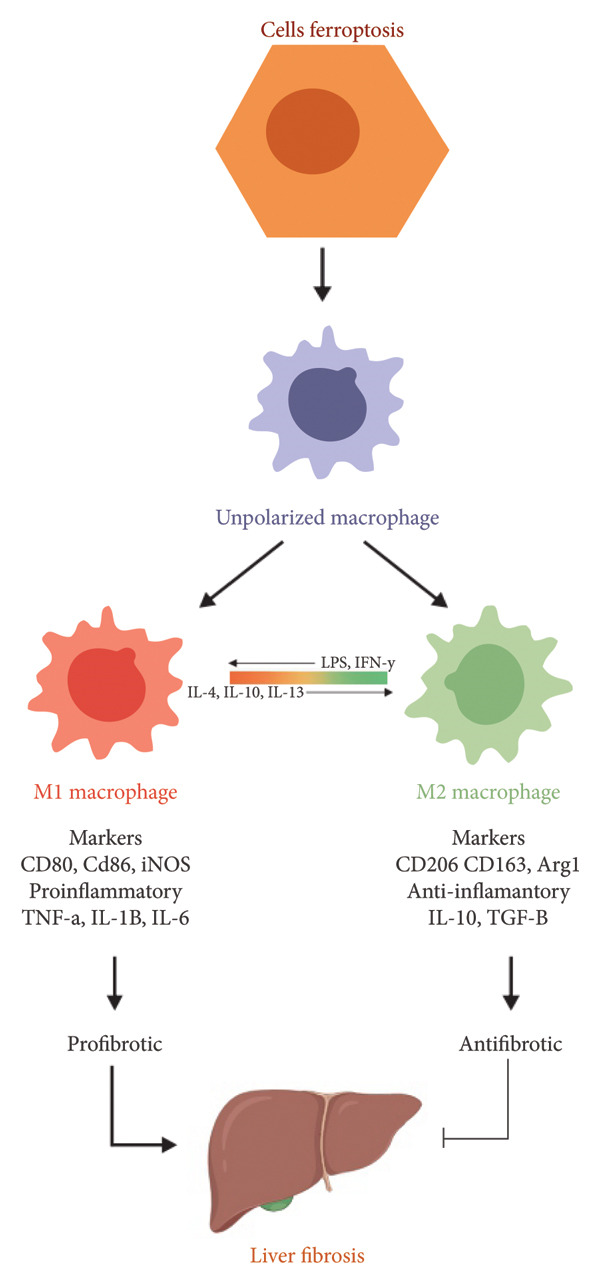
Polarization, secreting factor, and function of macrophage phenotype.

Macrophage M1 is a proinflammatory macrophage that is activated through the stimulus of LPS or interferon‐gamma (IFN‐γ), which secretes cytokines like IL‐1β, TNF‐α, and IL‐6 (Figure 3). The increase in ROS and cytokines is in response to hepatocyte cells that are damaged or experiencing cellular death, triggering the activation of HSC cells that produce collagen, leading to liver fibrosis. M2 macrophages are activated in response to the process of tissue repair through stimulation of IL‐4 and IL‐13 secretion (Figure 3), which release anti‐inflammatory and anti‐fibrosis cytokines [37]. M2 macrophages are one type of macrophage that is polarized toward an anti‐inflammatory or healing phenotype. M2 macrophages can be divided into several phenotypes based on the initiating signal, cell markers, and secreted factors.

M2 macrophages are further divided into several subtypes: M2a, M2b, M2c, and M2d, each induced by different stimuli and exhibiting distinct functional profiles. M2a is activated by IL‐4 and IL‐13 and promotes tissue repair and ECM deposition. M2b, induced by immune complexes and LPS, exhibits immunoregulatory functions. M2c is stimulated by IL‐10, TGF‐β, and glucocorticoids and is involved in anti‐inflammatory responses and tissue remodeling. M2d, also known as tumor‐associated macrophages, are induced by IL‐6 and adenosines and are associated with angiogenesis. In liver fibrosis, M2a and M2c are especially implicated in promoting fibrotic tissue formation through the secretion of TGF‐β and MMPs [36, 37].

Although M2 macrophages are often associated with tissue repair, certain M2 subtypes, particularly M2a and M2c, also play a significant profibrotic role. M2a macrophages, activated by IL‐4 and IL‐13, secrete TGF‐β and fibronectin, which can directly stimulate HSC activation and ECM deposition. M2c macrophages, while involved in matrix remodeling, express MMPs and TIMPs, which can shift the balance toward fibrosis depending on their ratio and chronicity of activation [38]. This dual role highlights the importance of context and temporal dynamics in macrophage‐driven fibrogenesis.

### 4.2. Molecular Interactions of Macrophages and Ferroptosis in Liver Fibrosis

Macrophages play a role in removing cell destruction debris from the body to maintain cellular balance [6]. According to several studies, both in vivo and in vitro macrophages that originate in endocrine tissues are more effective than those that originate in exocrine tissues at phagocytosis. Cells that experience damage or death will release certain signals known as DAMPs [33]. Endogenous chemicals known as DAMPs are generated by stressed, damaged, or dying cells and act as warning signs for the immune system. Common DAMPs involved in ferroptosis‐related inflammation include high‐mobility group box 1 (HMGB1), ATP, mtDNA, and heat shock proteins (HSP70, HSP90). These molecules interact with pattern recognition receptors (PRRs) such as TLRs, triggering inflammatory responses that perpetuate liver injury and fibrogenesis [39].

One of the main ways macrophages recognize cells that are about to die is through the expression of phosphatidylserine (PtdSer) on their surface [39]. In healthy cells, PtdSer is normally located on the inner layer of the cell membrane. However, when the cell undergoes apoptosis, PtdSer will be inverted and appear on the outer surface of the cell. In addition to the externalization of PtdSer as an “eat‐me” signal, macrophages express several receptors to recognize these apoptotic or ferroptotic cells. One such receptor is Stabilin‐1 (also known as Clever‐1), a scavenger receptor primarily found on alternatively activated (M2‐like) macrophages. Stabilin‐1 facilitates the clearance of apoptotic cells and contributes to immune tolerance and tissue remodeling. Its role in liver fibrosis is still emerging, but evidence suggests that Stabilin‐1 expression may enhance anti‐inflammatory signaling and support tissue repair, possibly making it a relevant modulator in the fibrotic response triggered by ferroptotic hepatocyte death [40]. Further studies are warranted to explore its specific function in the context of iron‐overload‐induced liver injury.

The iron can trigger the activation of HSCs and induce epithelial–mesenchymal transition, which is an important process in the pathogenesis of fibrosis [39]. Furthermore, iron overload can result in oxidative and inflammatory stress, activating M1 macrophages and sustaining profibrotic conditions via cytokines such as IL‐1β, TNF‐α, and TGF‐β. These cytokines promote HSC activation and ECM deposition [41, 42].

The summary of bioactive compounds and their antifibrotic mechanisms is presented in Table 1.

**Table 1:** A recent study of iron‐induced progression of liver fibrosis in an animal study.

Author	Inducer	Administered	Model	Results
[19]	Iron dextran	50 mg/kg BW, intraperitoneal, 7 weeks	Kunming mice	↑IL‐6, ↑TNF‐a, ↓SOD, ↓GSH, ↑MDA, ↑Caspase‐3. ↑Bax, ↓Bcl‐2, ↑Hyp, ↑Col1a1, ↑a‐SMA, ↑Fibrosis area
[22]	Iron dextran	Total 100 mg/kg BW, intraperitoneal, 5 times with a one‐day interval	Swiss mice	↑MDA, ↑Caspase‐3. ↑Bax, ↓Bcl‐2, ↑Hyp, ↑Col1a1, ↑a‐SMA, ↑Fibrosis area
[18]	A low‐fat diet with 2% carbonyl iron	Ad libitum, 26 weeks	C57Bl6 mice	↑Steatosis score, ↑inflammation score, ↑CD68, ↑CD86, ↑CCR2, ↑CCL2, ↑IL6, ↑collagen, ↑ASMA
[43]	Iron dextran	IP injection, 3x/week	C57BL/6 mice	↑ALT, ↑AST, ↑Fe, ↑Ferritin, ↑Total Fe, ↑α‐SMA, ↑COL‐1, ↑iNOS, ↑IL‐6, ↑TNF‐α, ↑Arg‐1, ↑IL‐10, ↑CD206 (3rd week), ↓CD206 (7th week), ↑Fibrosis area
[44]	Choline‐deficient L‐amino acid‐defined (CDAA) diet	CDAA diet vs. iron‐restricted CDAA diet for 4, 8, and 12 weeks	Male F344 rats	CDAA diet: ↑hepatic iron, ↑oxidative stress (8‐OHdG), ↑inflammation (CD68), ↑fibrosis (Sirius red); iron restriction ↓iron load, ↓fibrosis

Research conducted by Handa et al. [18] showed that dietary iron carbonyl causes a shift in macrophage polarization toward the proinflammatory M1 type, which exacerbates liver inflammation and accelerates fibrosis formation. Iron promotes activation of proinflammatory M1 macrophages, characterized by increased expression of M1 markers such as CD86. This activation contributes to chronic inflammation in the liver. On the other hand, iron inhibits the expression of M2 markers, such as arginase‐1 and CD163, which are important for anti‐inflammatory responses and tissue repair. Iron also decreases STAT6 phosphorylation required for M2 activation triggered by IL‐4, thereby inhibiting the transition of macrophages to an anti‐inflammatory state. Ferroptosis in hepatocytes leads to the release of DAMPs such as HMGB1, ATP, and mtDNA. These signals activate PRRs on Kupffer cells, including TLR4, biasing their polarization toward M1‐like states and promoting inflammation and fibrogenesis. IL‐1β, released by these activated M1 macrophages, further amplifies the immune response and HSC activation [18]. The mechanism is related to signaling pathways associated with increased ROS, such as NADPH oxidase (NOX), mitochondria, and NOX2, that are activated through NF‐κB and p38‐MAPK. Research conducted by Ren et al. [45] states that the decrease in GPX4 and the accumulation of iron produced through the Fenton reaction produce ROS that damage cells and trigger ferroptosis. In addition, activation of the IL‐6‐JAK‐STAT signaling pathway stimulates the production of hepcidin and ferroportin, causing conditions for the progress of ferroptosis due to iron excess in cells. Another cytokine released is TGF‐β, which triggers the activation and proliferation of HSC cells, which are activated into myofibroblasts to produce collagen. The released TGF‐β can also modulate the polarity of macrophages to differentiate into M2 macrophages in response to the repair process and protect damaged tissue (Table 2).

**Table 2:** Potential therapeutic targets of herbal compounds as anti‐fibrosis agents.

Target	Bioactive compound	Author
NF‐κB signaling pathway	Scoparone, 4‐hydroxy‐2(3H)‐benzoxazolone (HBOA), plumbagin, schisandrin B, p‐coumaric acid	[46–50]
Hedgehog signaling	Hesperetin derivative	[51]
PPAR signaling pathway	Schisandra‐B	[52]
TGF‐β1/Smad3 signaling	Gallic acid, ferulic acid, curcumin, anthocyanins, fucoidan, ligustroflavone, phlorizin, quercetin, kaempferol, tannins, chlorogenic acid	[53–64]
MAPK, Wnt, and PI3K/Akt signaling pathways	Tanshinone IIA, carvacrol, saponin, ligustrazine, and ferulic acid	[65–68]
IFN‐γ/STAT1/Smad7 signaling pathways	Glycyrrhizic acid	[69]
GSK‐3β pathway	Hastatoside	[70]
Gut–liver axis	Sodium alginate combined with oxymatrine	[71]
PDGFRβ signaling pathway	Salvianolic acid B	[72]
Other	Zingiber officinale, rice oil, policosanol, resveratrol	[19, 73–75]

Several research findings in this review indicate that the active ingredients contained in extracts have the potential and ability to address liver fibrosis. Various active ingredient mechanisms in managing liver fibrosis include the NF‐κB signaling pathway (5 studies: [46–50]), the TGF‐β1/Smad3 signaling pathway (12 studies: [53–64]), MAPK, Wnt, and PI3K/Akt signaling pathways (4 studies [65–68]), Hedgehog signaling [51], the PPAR signaling pathway [52], the IFN‐γ/STAT1/Smad7 signaling pathways [69], the GSK‐3β pathway [70], the gut–liver axis [71], the PDGFRβ signaling pathway [72], and other mechanisms not specifying signaling pathways ([19, 73–75]).

## 5. Molecular Pathway

### 5.1. NF‐κB Pathway and Ferroptosis

**Figure 4:**
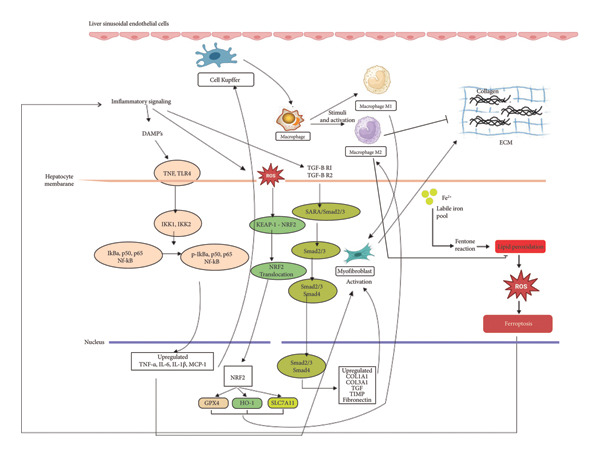
The molecular pathway of activation of macrophages in the progression of liver fibrosis.

The nuclear factor‐kappa B (NF‐κB) signaling plays a crucial role in regulator of inflammatory responses and cellular survival. This signaling cascade is activated by various stressors, including oxidative stress, cytokines, and infections (Figure 4). After migrating to the nucleus, NF‐κB alters the transcription of genes related to apoptosis, inflammation, and the immune system [76].

Emerging studies indicate a complex interplay between NF‐κB signaling and ferroptosis, a form of regulated cell death characterized by iron‐dependent lipid peroxidation. NF‐κB can exert both protective and promotive effects on ferroptosis, depending on the cellular context. On the one hand, NF‐κB activation upregulates heme oxygenase‐1 (HO‐1) and ferritin heavy chain (FTH1), which counteract oxidative stress and inhibit ferroptosis. On the other hand, prolonged NF‐κB activation can increase the expression of proinflammatory cytokines like tumor necrosis factor‐alpha (TNF‐α) and interleukin‐6 (IL‐6), exacerbating lipid peroxidation and promoting necroptotic cell death [77]. Additionally, NF‐κB signaling is implicated in modulating iron metabolism, a critical determinant of ferroptosis susceptibility. The pathway influences the expression of genes involved in iron homeostasis, such as transferrin receptor 1 (TFR1) and ferroportin (FPN1). Dysregulation of these factors can lead to iron accumulation, thereby sensitizing cells to ferroptotic stress [77].

The NF‐κB pathway plays a key role in modulating HSC activation, which contributes to liver fibrosis and its progression to cirrhosis. Targeting this pathway may offer a promising therapeutic strategy for mitigating liver fibrosis [78]. Scoparone, a bioactive compound extracted from *Artemisia capillaris* Thunb, has been shown in a study by Liu et al. [46] to alleviate hepatic steatosis, apoptosis, inflammation, and fibrosis in methionine‐ and choline‐deficient (MCD)‐induced NASH murine models. Treatment with scoparone has been found to suppress NASH‐related inflammation and immune responses triggered by LPS in macrophages by inhibiting TLR‐4/NF‐κB signaling. Additionally, other bioactive compounds, including hydroxy‐2(3H)‐benzoxazole (HBOA), *plumbagin*, *schisandrin* B, and *p*‐coumaric acid, have demonstrated potential antifibrotic effects in the liver by modulating inflammatory pathways, particularly through NF‐κB signaling inhibition and HSC deactivation [47–50].

### 5.2. TGF‐β Pathway and Liver Stellate Cell Activation

TGF‐β serves as a pivotal regulator in liver fibrosis, primarily by modulating HSC activation. Under physiological conditions, HSCs remain in a dormant state, where they store vitamin A and contribute to the maintenance of ECM homeostasis. However, when a liver injury occurs, TGF‐β signaling initiates a series of molecular events that drive HSC activation, ultimately leading to their differentiation into myofibroblast‐like cells. These activated HSCs excessively produce ECM proteins, including collagen, which play a crucial role in fibrotic tissue formation [79].

TGF‐β regulates fibrogenesis through both canonical and noncanonical signaling pathways (Figure 4). In the canonical pathway, TGF‐β ligands bind to type I and type II receptors, leading to the phosphorylation of SMAD2 and SMAD3 [80]. These phosphorylated SMADs interact with SMAD4 and subsequently migrate into the nucleus to control the transcription of profibrotic genes [81]. This signaling cascade plays a crucial role in liver fibrosis by increasing the expression of connective tissue growth factor (CTGF) and alpha‐smooth muscle actin (α‐SMA), which are essential markers of HSC activation. Apart from the SMAD‐dependent mechanism, TGF‐β also activates noncanonical pathways, including mitogen‐activated protein kinases (MAPKs), phosphoinositide 3‐kinase (PI3K)/Akt, and Rho‐like GTPases [82]. These alternative signaling routes further drive HSC activation and contribute to fibrosis development by promoting cell proliferation, survival, and migration. Additionally, crosstalk between TGF‐β and other signaling networks, such as NF‐κB and Wnt/β‐catenin pathways, introduces additional complexity to fibrosis regulation, highlighting the intricate molecular mechanisms underlying liver fibrosis progression.

Given the important role of the TGF‐β1 signaling pathway in the process of inflammation and the formation of liver fibrosis, it is possible that the signaling pathway could be a therapeutic target in reducing the effects of liver fibrosis [83]. Several alternative therapies using herbal ingredients have been researched by targeting the TGF‐β1/Smad signaling pathway. Research conducted by [84–89] with the use of active ingredients derived from herbal extract products such as gallic acid, ferulic acid, curcumin, anthocyanins, fucoidan, ligustroflavone, phlorizin, quercetin, kaempferol, tannins, and chlorogenic acid showed results that potentially reduce the effects of fibrosis. Curcumin, a bioactive compound extracted from *Curcuma longa*, has demonstrated potential in mitigating liver damage, alleviating oxidative stress, and inhibiting fibrosis while normalizing MMP‐9 and MMP‐2 activity. Furthermore, curcumin has been shown to regulate protein expression levels of NF‐κB, IL‐13, IL‐10, TGF‐β1, CTGF, Col‐I, MMP‐13, and Smad7, contributing to its protective effects on liver health. On the other hand, curcumin decreased the phosphorylation of JNK and Smad3. In addition, curcumin treatment decreased protein and mRNA levels, α‐SMA, and Smad3. Curcumin normalized GSH, NF‐kB, JNK‐Smad3, and TGF‐β1/Smad3 pathways, causing a decrease in activated HSCs, thus producing an antifibrotic effect [56].

### 5.3. Nrf2–GPX4 Pathway in Liver Protection Mechanism

Nuclear factor erythroid 2‐related factor 2 (NRF2) is a transcription protein that regulates cellular responses to damage [90]. NRF2 plays a role in activating several genes involved in antioxidant metabolism, including GPX4, which has a crucial function in preventing ferroptosis. NRF2 functions as a regulator of cellular defense against oxidative stress by increasing GSH production, which is necessary for GPX4 activity to reduce lipid peroxidation that can trigger ferroptosis (Figure 4). Research conducted showed that NRF2 activation can increase GPX4 expression and inhibit lipid peroxidation in hepatocyte damage triggered by oxidative stress. In a CCl4‐induced liver damage research model, the increased expression of NRF2 and GPX4 showed a reduction in lipid peroxide accumulation and a decrease in the level of ROS in the liver. In addition, NRF2 also plays a role in regulating iron homeostasis through regulating the expression of related genes such as FTH1 (ferritin heavy chain 1), FPN1 (ferroportin 1), and FTL (ferritin light chain) that reduce iron stress in cells and inhibit the role of iron in exacerbating ferroptosis [91].

NRF2 regulates several metabolic pathways that contribute to the inhibition of ferroptosis. One of the main pathways involved is the Cystine/GSH/GPX4 pathway, where NRF2 activates the expression of SLC7A11 (xCT), which plays a role in the transportation of cystine into the cell to be converted into cysteine and used for GSH biosynthesis [92]. GSH is required for GPX4 activity in reducing lipid peroxides. A decrease in GSH levels or GPX4 inactivation can trigger ferroptosis. Research conducted by Zhao et al. [93] stated that the Nrf2–GPx4 axis plays an important role in the repair of liver damage by ferroptosis in acute liver damage. The same researcher also showed that Bicyclol treatment was shown to regulate the Nrf2–GPx4 pathway, which helps reduce liver damage caused by CCl4 by inhibiting ferroptosis. The study also showed that CCl4 caused a significant decrease in GPx4 expression and increased iron accumulation in the liver. The treatment effect showed improvement by restoring the reduced GPx4 levels, increasing the expression of xCT (a cysteine‐transporting protein involved in GSH production), and reducing iron and ROS levels in liver tissue, as well as increasing Nrf2 expression [94].

### 5.4. Therapeutic Potential and Clinical Translation

While ferroptosis inhibitors such as ferrostatin‐1 and liproxstatin‐1 have shown promise in preclinical models, their clinical application remains experimental. Deferasirox, a clinically approved iron chelator, has been shown to reduce hepatic iron burden in thalassemia patients and may indirectly mitigate ferroptotic injury. Similarly, statins and natural antioxidants such as vitamin E have demonstrated ferroptosis‐modulatory effects through the activation of Nrf2 and GPX4 pathways [95].

However, several barriers limit their translation. Oral bioavailability, hepatic targeting, and the dynamic interplay between macrophage subtypes complicate consistent therapeutic outcomes. Emerging strategies, including nanoparticle delivery systems and dual‐action compounds that target both immune polarization and ferroptosis, may provide a more comprehensive approach to preventing fibrosis progression in iron‐overload conditions.

## 6. Conclusion

In summary, the NF‐κB and TGF‐β pathways play pivotal roles in the regulation of inflammation, cellular survival, and fibrosis. The intricate balance between these pathways determines the fate of cells in various pathological conditions, including ferroptosis and liver fibrosis. NF‐κB signaling exhibits both protective and deleterious effects on ferroptosis, while TGF‐β remains a critical driver of HSC activation and fibrosis development. A deeper understanding of the molecular interplay between these pathways could open new avenues for therapeutic interventions. Targeting specific components within these cascades offers promising prospects for treating diseases driven by oxidative stress, chronic inflammation, and fibrotic remodeling. Future research should focus on refining targeted therapies to selectively modulate these pathways, minimizing adverse effects while maximizing clinical benefits.

## Conflicts of Interest

The authors declare no conflicts of interest.

## Funding

This research did not receive any specific grant from funding agencies in the public, commercial, or not‐for‐profit sectors.
